# Using transfer learning approaches to predict RNA-Seq gene expression data for cancer classification

**DOI:** 10.3389/frai.2026.1790249

**Published:** 2026-03-19

**Authors:** Waqas Haider Bangyal, Adnan Ashraf, Zia Ul-Qayyum, Meshari Alazmi, Asma Abdullah Alfayez

**Affiliations:** 1 Department of Computer Science, Kohsar University, Murree, Pakistan; 2 Department of Computer Science, Government College Women University Sialkot, Sialkot, Pakistan; 3 Department of Computer Science, FAST - National University of Computer & Emerging Sciences, Islamabad, Pakistan; 4 College of Computer Science and Engineering, University of Ha'il, Ha'il, Saudi Arabia; 5 King Abdullah International Medical Research Center, Riyadh, Saudi Arabia; 6 King Saud bin Abdulaziz University for Health Science, Riyadh, Saudi Arabia; 7 Ministry of National Guard Health Affairs, Riyadh, Saudi Arabia

**Keywords:** cancer, gene expression data, ResNet, RNA-Seq, transfer learning

## Abstract

There is a great need to categorize cancer types for early cancer detection and treatment. RNA-Seq data is essential for getting insight into the differentially expressed genes. Due to its high dimensionality and complexity, performing an analysis on RNA-Seq data is quite challenging. In the past, RNA-Seq data were analyzed for a single cancer type as a two-class problem (either positive or negative) and did not contain information from other classes of cancer types. To classify different cancer types and discover the most promising genes, RNA-Seq data for different types of cancer should be examined. Multiple repositories offer RNA-Seq-based cancer types data. The present study incorporates a dataset from the Mendeley repository for classification. RNA-Seq values are then converted to their respective 2D images using some transformations. The classification problem is handled by five Transfer Learning (TL) algorithms (VGG16, VGG19, Resnet50, Resnet101, and Resnet152). Four different splitting strategies are applied for each classifier presented in the results and discussion section. A comparative analysis is also carried out with and without data augmentation. Results show that classifiers perform best at a split of 70-30. VGG16 attained the best position on overall results by achieving an accuracy of 95%. Hence, VGG16 is the leading TL algorithm for classification among all the accessible models and is not difficult to execute and easy to comprehend.

## 1 Introduction

**Figure 1:**
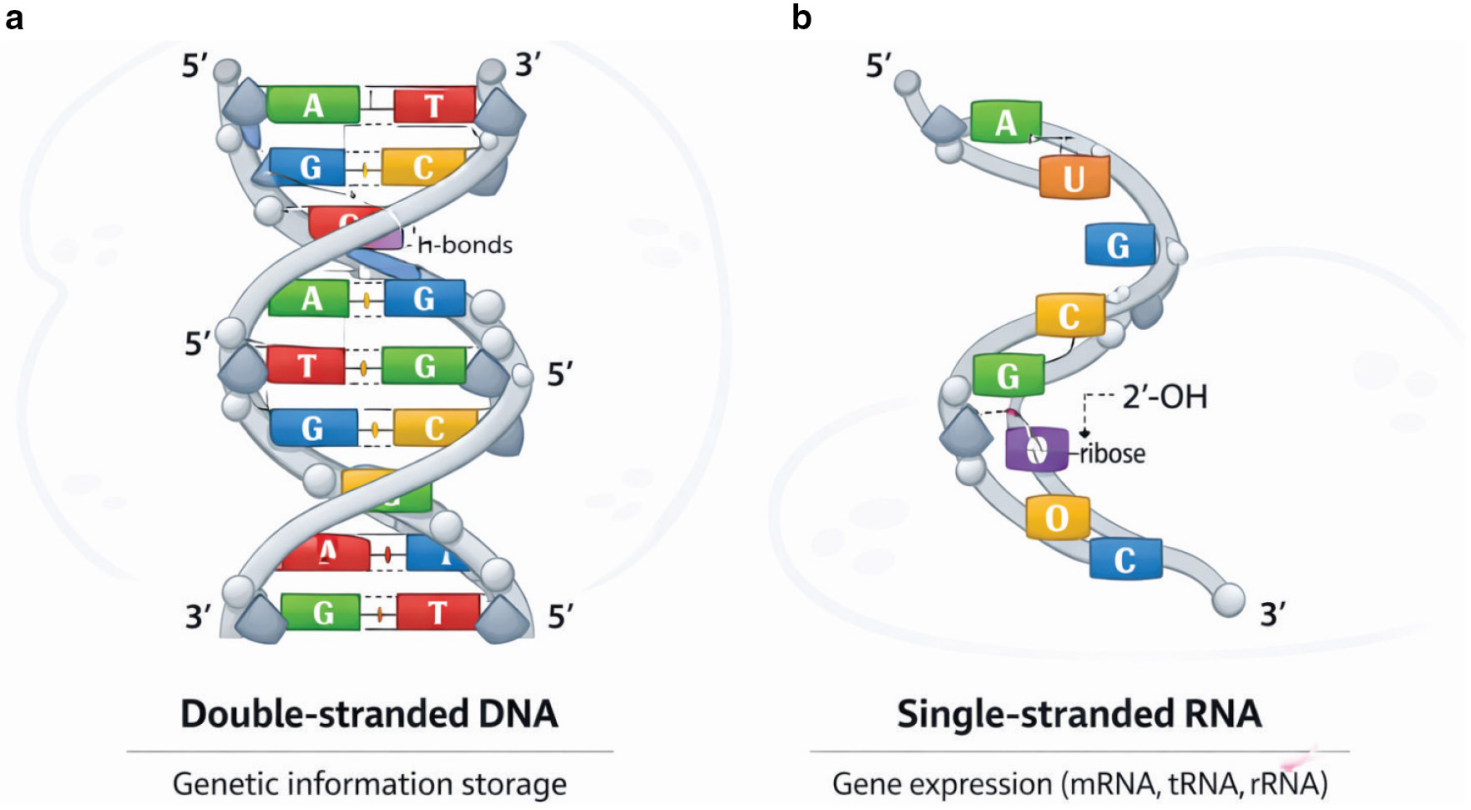
**(a, b)** Structures of DNA and RNA, A: adenine, G: guanine, T: thymine, and C: cytosine.

Cancer is the most dreadful and severe type of disease worldwide (Khalifa et al., 2020). Various cancer types may reside in the human body, and in many cases, they certainly cause death to their patients (Ali et al., 2021). Anomalous behavior and unusual division of some cells in the body cause cancer. These cells also harm other nearby cells, and a clot or tumor forms. Early detection and accurate prediction of the survival of patients may lead to the best treatment and may save a patient's life (Hassanzadeh et al., 2016). However, its early detection is complicated because its death rate is very high, and its cells are disordered (Xiao et al., 2018a; Eralp and Sefer, 2024; Yao et al., 2025). RNA-Seq data is helpful in cancer detection and accurate prediction. For this purpose, RNA-Seq data is considered and analyzed, which provides large gene expression data for the prediction and classification of multiple diseases (Siddaway et al., 2025; Tuncer et al., 2022). Figure 1a shows the structure of DNA, and Figure 1b shows the structure of RNA.

A popular technique in bioinformatics that offers normalized and fewer noisy data instances and detects new isoforms and transcripts for prediction and classification from gene expression data is called RNA-Seq (Goksuluk et al., 2019). RNA-Seq analysis is of high importance for getting insight into gene activity and has led to significant research in the biomedical field in this era (Dalla-Torre et al., 2025). The most basic capacity of transcriptome profiling is determining whether differentially expressed genes are present or identifying variations in genes at various levels. Identification and quantification of both tasks in a single step can be performed using RNA-Sequencing (Muhammad et al., 2025; Conesa et al., 2016). RNA-Seq Data is promptly accessible in various datasets and is generally used to categorize diseases like Colon Adenocarcinoma (COAD), Kidney Chromophobe, Breast Invasive Carcinoma (BRCA), etc. (Urda et al., 2017). However, analyzing RNA gene expression data is complex due to its high dimensionality, complexity, and duplication in feature values (Danaee et al., 2016). Some feature selection algorithms are required to work with this high-dimensional data (Ashraf et al., 2025). Consequently, a requirement for automated feature extraction is needed, and Machine Learning (ML) and Deep Learning (DL) models come.

There are a few constraints of ML-based methods for choosing promising features from biomedical images for classification purposes (Cruz and Wishart, 2006; Ahmed et al., 2022). Hence, Deep learning (DL) emerged as a solution to this problem. Deep Learning is a strategy that eliminates the intermediate feature extraction steps and attempts to zero in on making a determination based on the input data. For that reason, it is additionally named “automated Feature Engineering” (Gad et al., 2018). DL has revolutionized the field of computer vision (Rong et al., 2019). DL works in layers, taking input, processing, analyzing, and identifying underlying patterns in data. DL is applied for voice recognition, visual object detection (He et al., 2023). Deep Learning is effectively utilized in multiple research areas, including bioinformatics, medical image analysis, and graphical data handling. A Convolutional Neural Network (CNN) is a DL model for working with large-scale image data, utilizing weighted connections, subsampling, and localized connectivity procedures. CNN removes the irrelevant features and decreases NN's complexity (Santamato and Marengo, 2025; Ashraf et al., 2021; Gezici and Sefer, 2024).

CNN performs admirably on big data, but it struggles on small datasets, especially when it comes to classification and prediction (Ashraf et al., 2023). Implementing the core concept of transfer learning can increase classification accuracy with minimal datasets, and employing the learned parameters can improve the effectiveness of CNN models (Bangyal et al., 2018; Ismail et al., 2025). In this view, a model is trained on a large dataset, features are extracted, and the extracted information is then applied to a more limited dataset [44]. Transfer learning has been shown to boost model performance in numerous studies (Mahajan and Sharma, 2024). TL is a method of solving problems by transferring what has been learned by solving comparable problems in the past (Zhou et al., 2020). In areas where machine learning has proven useful, such as text emotion classification (Alam et al., 2025), picture analysis and categorization (Sakirin and Said, 2025; Abbasi-Vineh et al., 2025), human movement detection (Rani and Kumar, 2025), software flaw identification (Boovaraghavan et al., 2025), forensics (Fattahi et al., 2024), etc., it has been put to use.

The differential analysis is the main piece of RNA-Seq analysis. Traditional differential analysis strategies ordinarily match the tumor tests to the normal samples, both from a similar cancer type. Such a strategy would fail to separate cancer types since it needs information on other cancer types. To better understand the causes for different cancer types, a detailed analysis of RNA-Seq data is needed (Blawski et al., 2024). For the identification of the most promising genes, most investigations attempted to identify the differentially expressed genes. Therefore, the construction of a technique that can incorporate information from different cancer types into the analysis is necessary (Gholizade et al., 2025; Thakuria et al., 2024). Five transfer learning algorithms are applied for classification in this study. This method will be applied to RNA-Seq images of numerous cancer types and will classify them accordingly. These transfer learning algorithms are carried out in this study with various data-splitting techniques, with and without augmentation. Furthermore, the objectives of this research work have been detailed in the following:

- To design a new method to discover promising genes from data.
- To find the most accurate method to show improvement compared with the previous study on tumor type classification.
- To examine the most accurate method with the best results among all available methods.

The remainder of the study is organized as follows. Section 2 includes the related work. Section 3 describes the material and technique. Section 4 presents trial results, while the discussion is presented in Section 5, and Section 6 concludes the study.

## 2 Related work

Ryvkin et al. (2014) introduced a new mathematical technique for the classification of RNA by Analysis of Length (CoRAL). The authors took small RNA sequence datasets and sequenced them, followed by numerous preprocessing steps, i.e., a dataset is passed to manage three adapter sequences, and a FASTQ record is created. By coordinating with a reference record, reads are aligned, and results are put away in a SAM document. CoRAL concentrates significant features and orders different kinds of RNA sequences. This strategy not only groups small RNA Sequences; rather, it provides better guidance to the research community.

To diagnose thyroid cancer, two approaches, Denoising Autoencoders (DAE) and SDAE, were proposed in Kondaveeti and Edupuganti (2020). This strategy for feature selection retrieves the most useful features from RNA-Seq data, which are then fed into the model for better thyroid diagnosis. DAE consists of noisy data to get more useful data by avoiding overfitting problems. This methodology has been compared to CPA and KPCA and provided near-best results, and with better resources, it can yield the best results.

To predict the disease state from the given RNA-Seq data, a Graph Attention Network was proposed by Singh and Singh (2020). This model is tested on MS patients and healthy individuals. The trained graph attention model is used to pick significant transcripts and genes for MS prediction. This methodology emphasizes disease prognosis and how an individual's data can be used to calculate disease survival. The proposed methodology produced more accurate findings compared to state-of-the-art approaches such as convolutional networks, random forests, and multilayer perceptrons.

Khan and Rodrigues (2019) also presented an original methodology for the grouping of breast cancer. Two breast cytology image datasets were utilized in this review. These pictures were then preprocessed and underwent some changes for augmentation. The authors utilized a transfer learning approach utilizing three distinct CNN models, i.e., GoogleNet, ResNet, and VGGNet. These three CNN models were pre-trained on the ImageNet dataset. Features were extracted utilizing these three pre-trained models independently and joined at completely associated layers to distinguish malignant from benign using max pooling. The proposed structure showed preferable outcomes overall.

Xiao et al. (2018b) set up another deep learning-based multi-model ensemble approach that utilizes five ML models. This proposed technique is applied to three diseases, including LUAD, Stomach Adenocarcinoma (STAD), and BRCA. This technique is carried out with the goal that every classifier on given information gets forecasts exclusively and afterward, utilizes these forecasts in a multi-model ensemble approach utilizing deep learning.

Wu and Hicks (2021) utilized different AI calculations to group triple-negative breast cancer from non-triple-negative breast cancer. For experiments, RNA-Sequencing and gene expression data were downloaded from TCGA for 110 triple-negative breast cancer samples and 992 non-triple-negative samples. Support Vector Machines (SVM), K-closest neighbor (KNN), Naïve Bayes (NB), and Decision Tree (DT) were used as grouping models of AI. On account of the high data dimensionality, an additional stage named feature selection was performed to get the most relevant features before characterization. SVM performed better compared to all other models. Moreover, the accuracy values of the models were 90%, 87%, 85%, and 87%, respectively.

McDermaid et al. (2018) presented an ML-based apparatus named Gene Expression Quality Control (GeneQC) for the assessment of the reliability of expression levels in the precise RNA grouping dataset. The 95 RNA sequencing datasets were used from an aggregate of 7 plants and animal species. Ultimately, GeneQC groups the classification of the read alignment of each and every genome.

Khomsay et al. (2019), and Han et al. (2025) put up a new method for detecting cough (Productive and non-Productive) by using PCA with Deep Learning Networks (DLN) with the use of TensorFlow. This method is applied to eight different samples of cough by eight different individuals. First of all, the sound generated is captured by the Fast Fourier Transform using signal spectra. Then, PCA extracts the most relevant signals and sends them to the DLN model to work on by using its TensorFlow Library. The proposed methodology is compared with simple DLN. Results showed that PCA with DLN outperforms in accuracy, error rate, and processing time compared to simple DLN.

Kondaveeti and Edupuganti (2020) used the Transfer Learning approach to classify skin cancer. The Ph2 dataset was used in the study for training purposes. AlexNet is used here as a transfer learner pre-trained on ImageNet. The proposed methodology worked in three phases. The Softmax layer replaced the last classification layer of AlexNet to classify three skin lesions. The weights of the changed model are then fine-tuned to get better accuracy. Finally, augmentation was performed to overcome the problem of overfitting. The proposed methodology achieved better accuracy and outperformed when compared with the state-of-the-art in the literature.

## 3 Material and method

### 3.1 Dataset

The Gene expression dataset utilized in this study is accessed from Kakati et al. (2022). This dataset incorporates gene expression advantages of 5 unique types of cancer, i.e., Breast Invasive Carcinoma (BRCA), Kidney Renal Clear Cell Carcinoma (KIRC), Uterine Corpus Endometrial Carcinoma (UCEC), Lung Adenocarcinoma (LUAD), and Lung Squamous Cell Carcinoma (LUSC). Brief insights concerning these infections are given in the preceding sections.

#### 3.1.1 BRCA

BRCA, abbreviated as Breast Invasive Carcinoma (Alruwaili and Gouda, 2022), is the most awful and noxious kind of disease in women. Two gentle malignant growth types, i.e., ILC and IDC, play 90% in developing BRCA in the body, and just IDC is the overall sort and plays 80% of it.

#### 3.1.2 KIRC

Kidney Renal Clear Cell Carcinoma, likewise referred to as KIRC, is the broadest type of malignant growth with a high death proportion worldwide (Nasir et al., 2022). Kidney malignant growth is normal, and KIRC generally turns into the cause of renal disease in 70%–80% of patients.

#### 3.1.3 LUAD

Lung Adenocarcinoma, likewise called LUAD, is the standard type of disease. LUAD essentially comprises 40% of all Lung malignant growth types, and by and large, it seldom affects non-smokers (Maqbool et al., 2025). By and large, LUAD is experienced coincidentally and extends gradually than other kinds of cellular breakdowns in the lungs.

#### 3.1.4 LUSC

Lung Squamous Cell Carcinoma is also known as LUSC. LUSC is a runner-up of Lung awful diseases and is common in tobacco smokers for the most part. Smoke particles in the air spread LUSC malignant growth, making their home normally in the center of the lung (Rahman et al., 2022).

#### 3.1.5 UCEC

Uterine Corpus Endometrial Carcinoma, otherwise called UCEC, is a repetitive pre-birth toxic malignant growth that can't be recognized at its initial stages (Wang et al., 2024). It is the most well-known sort of disease in women. It has a high demise proportion due to the lack of access to data on its biomarkers for early discovery and treatment.

Table 1 presents a number of interesting observations with regard to cancer types in the dataset.

**Table 1:** Dataset description.

**Sr#**	**Cancer type**	**Number of instances**
01	Breast invasive carcinoma	878
02	Kidney renal clear cell carcinoma	537
03	Lung adenocarcinoma	162
04	Lung squamous cell carcinoma	240
05	Uterine corpus endometrial carcinoma	269

### 3.2 Data pre-processing

Several steps were performed to change over gene expression profiling 1D information into an image configuration to be offered to Neural Networks and other classification models. In pre-processing stage, we changed mathematical 1D information over to 2D pictures information with the accompanying advances: Loaded the information into memory, then, at that point, we standardized the qualities from the scope of (0 to 24,248) to (0 to 255) territory with the assistance of equation 1 where 255 is the most noteworthy worth of a picture in pictures range, and 24,248 is the most noteworthy worth in the gene expression dataset. In addition, at the last advance, 971 features were changed over to [32 × 32] pixel pictures by affixing nearly zeros at the last of images. Reshaping 971 gene features into a [32 × 32] grid with zero-padding is only an input-formatting step to enable TL (not a biological spatial mapping), while preserving original feature values with minimal padding impact, also, this progression's result, we have 2,086 pictures of size [32 × 32].


$$\begin{array}{l} \text{Pixel}=\mathrm{Round}\;\left(\frac{\text{CellValue}\times 255}{24,248}\right) \end{array}$$ (1)

#### 3.2.1 Feature engineering

The feature engineering task is further divided into two steps.

##### 3.2.1.1 Feature extraction

**Figure 2:**
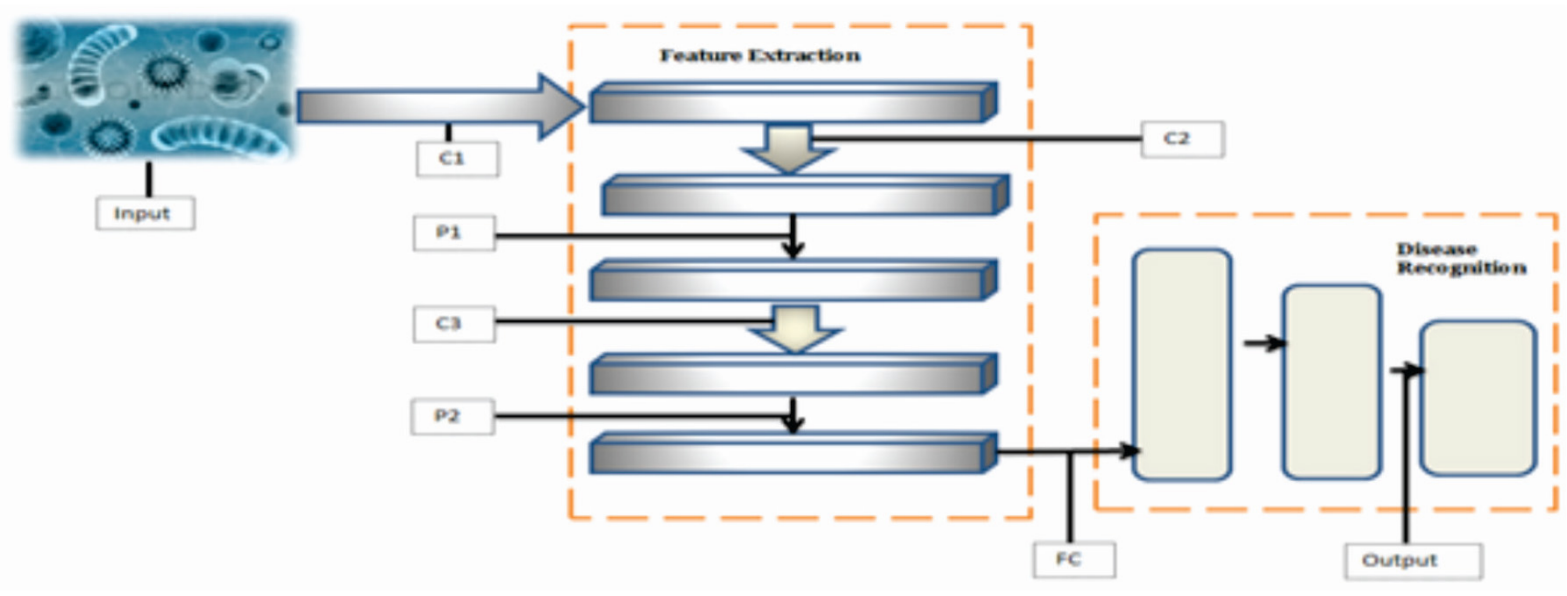
Architectures of VGG-16 and VGG-19.

Working with RNA-Seq with a variety of features makes it more difficult to work on and build a classification model (Ashraf et al., 2025). For this purpose, in this study, feature extraction on RNA-Seq data is performed. Feature extraction is a technique that allows us to work with subsets of features as well as to prevent the problem of over-fitting. Feature extraction also improves model performance as a reduced number of samples, or in this case, genes, are there to be analyzed and tested. There are multiple feature extraction techniques used by researchers in their research work, i.e., PCA and Variational Auto Encoders, filter, wrapper, and embedded methods (Pal et al., 2025). As represented in Figure 2.

##### 3.2.1.2 Feature selection

The selection of the most relevant features from the given dataset is very important. Therefore, after extracting features from different deep learning algorithms, the selection of the best features from that feature set is performed. The feature selection process increases understanding of datasets, improves results and accuracy, and helps in eliminating irrelevant features that may cause high computations. EC algorithms are being used in almost every field of study for feature selection purposes. Different EC approaches, like the Firefly Algorithm, are implemented in different studies. The Genetic Algorithm for feature selection enhances the performance of classifiers. In this thesis, the Genetic Algorithm is applied for feature selection (Pal et al., 2025).

#### 3.2.2 Data augmentation

Dimensionality expansion implies synthetically expanding the size of features to defeat the possibility of overfitting the model (Abbasi-Vineh et al., 2025). Different expansion parameters are accessible to build the size of the information. We can utilize parameters as per our own prerequisites. In this review, level flip, zoom range, vertical flip, and shear are utilized. Also, an extensive comparative investigation of information with and without augmentation strategies is given in the results and discussion.

### 3.3 Transfer learners

In this review, five Transfer Learning models are executed for characterization purposes. A brief insight regarding each model is summarized below.

#### 3.3.1 VGG

**Figure 3:**
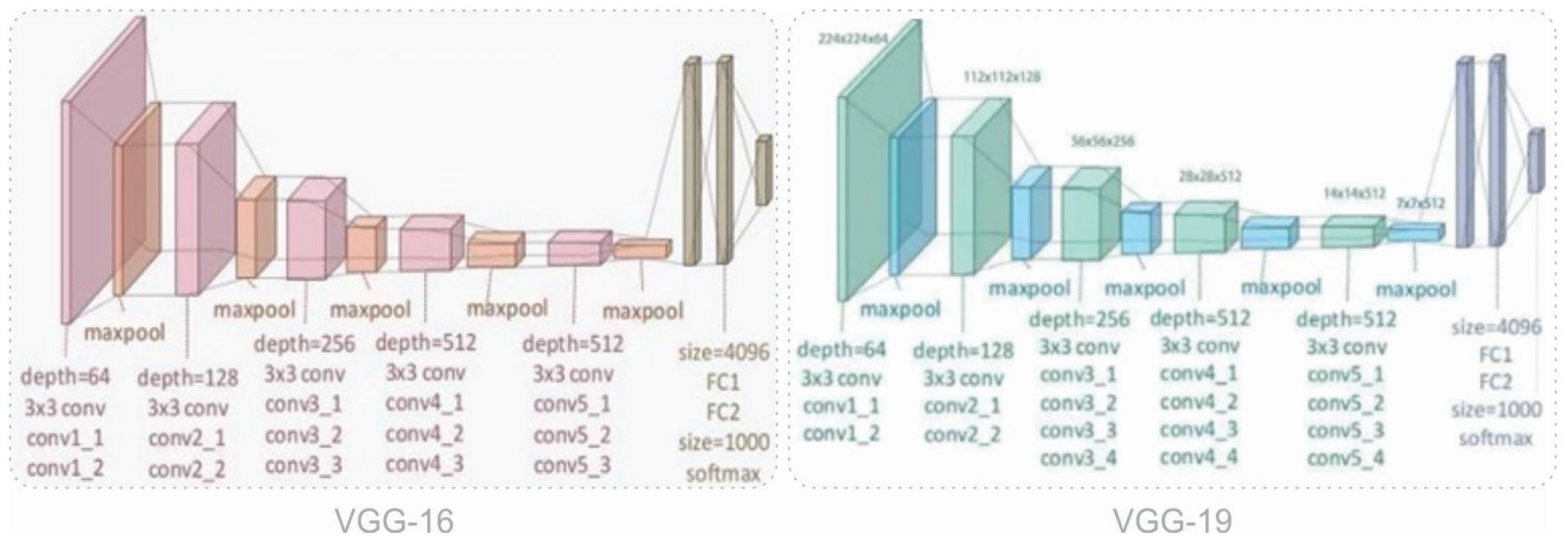
Architectures of VGG-16 and VGG-19.

Transfer learning (TL) models transfer knowledge from one task to another task. A model developed and used by some people for a task and utilized for another but similar tasks by others is called a pre-trained model. VGG16 is a well-known model utilized in TL models for feature extraction and classification. VGG16 is a CNN approach used to reduce the number of features required for convergence. It has 16 layers in its design and uses roughly 138 million parameters for classification tasks. Pre-training of the VGG model is done on the ImageNet dataset (Simonyan and Zisserman, 2014). VGG-16 and VGG-19 architectures are represented in Figure 3.

VGG19 is a variant of VGG16, and the number of layers used in this architecture is 19. There are 16 convolutional layers and three fully connected layers in this architecture. It is generally implemented as a pre-trained model, and the depth of VGG-19 has been tremendously enhanced. It extracts features in a more effective way with the help of max-pooling for downsampling and by making modifications to ReLU.

#### 3.3.2 RESNET

**Figure 4:**
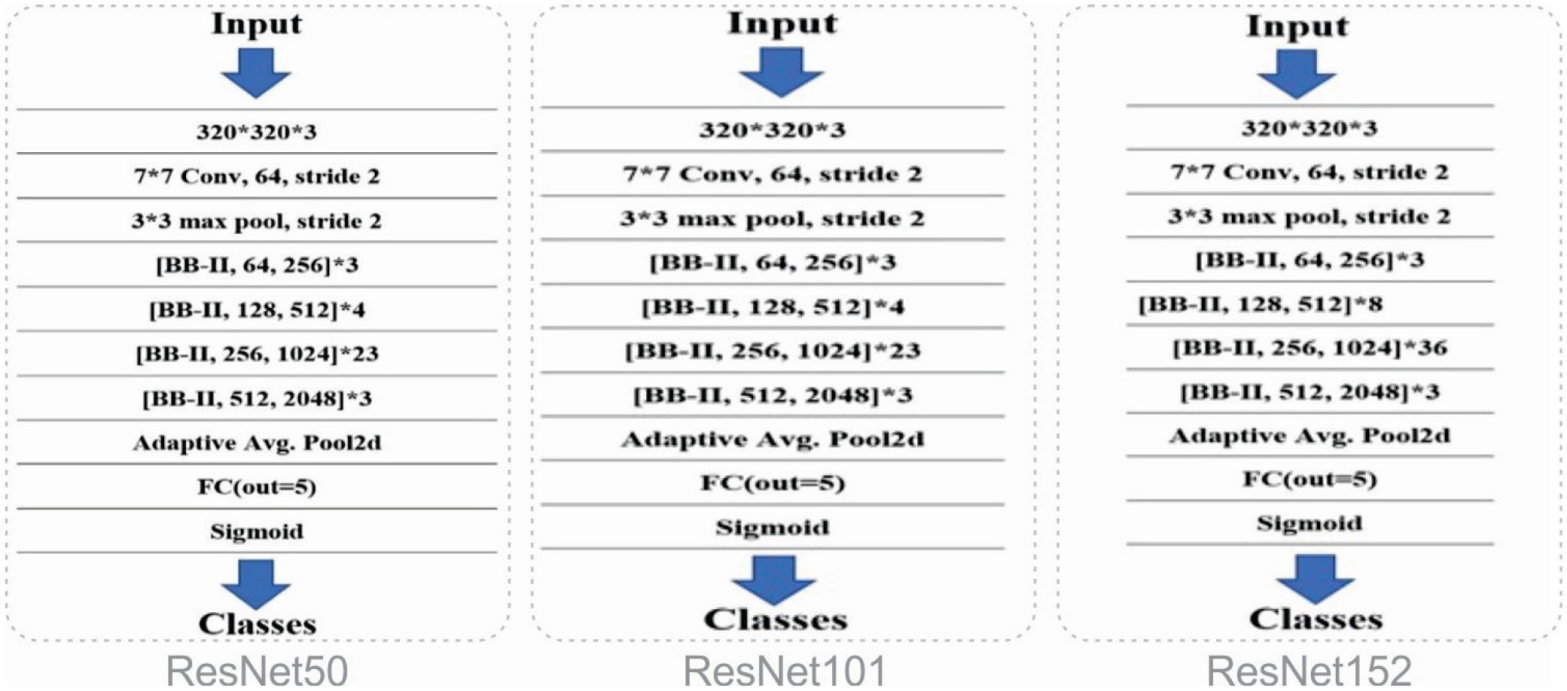
Architectures of ResNet50, ResNet101, and ResNet152.

esNet, short for Residual Network, was proposed in 2015 by researchers at Microsoft. The fundamental reason behind proposing and presenting a new design, while CNN had already existed at that point, was vanishing gradient problem. Adding profundity to the model backpropagation hits the slope, and performance degrades drastically [90]. Subsequently, this ResNet model presented skip associations that imply which layers add to reducing the model's performance are skipped by this association design. It accomplishes extremely noteworthy outcomes for image-processing tasks (Sharifuzzaman et al., 2025). ResNet structures are utilized for non-computer-vision tasks alongside image classification. This architecture has roughly 23 million parameters to be prepared. There are numerous ResNet models accessible. ResNet50 is a variant of ResNet that is 50 layers deep. There are 48 convolutional layers, one max pool, and one average pooling layer in the design. The most well-known CNN architecture is being utilized. ResNet101 is one more variant of ResNet, which is twice as deep as ResNet50. It includes 101 layers and can be utilized to classify many classes easily. Figure 4 shows the architectures of ResNet50, ResNet101, and ResNet152.

### 3.4 Proposed system design

The dataset used in this study for multiple tumor types with 2,086 samples and 971 features in the dataset, and the last column represents the tumor category. Tumors classified in this study are Lung Squamous Cell Carcinoma, Kidney Renal Clear Cell Carcinoma, Breast Invasive Carcinoma, Uterine Corpus Endometrial Carcinoma, and Lung Adenocarcinoma. RNA-Seq values of each cancer type are then converted to 2D images through preprocessing. In the preprocessing phase, the first step, numeric gene values, are normalized to the image range using Equation 1. In the next step, each sample is transformed into an image of size 32 × 32 by padding some zeros to the end of the images. A total of 2086 images were generated as an output of this process. These images are passed to five TL models, i.e., VGG16, VGG19, ResNet50, ResNet101, and ResNet152.

#### 3.4.1 Training and testing strategy

**Figure 5:**
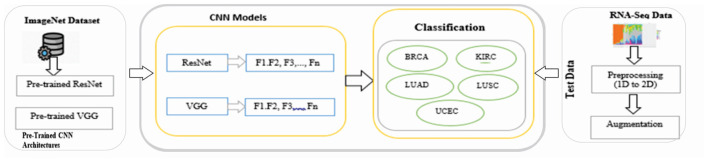
Workflow diagram of the proposed system design strategy.

This study utilizes four different splitting methodologies to gauge precision and break down various procedures on our proposed design. 1st procedure is the point at which we pass half the information to training and half the information to testing. The subsequent procedure infers 60% information for training and 40% for testing. 3rd procedure makes an order on 70% preparation and 30% test information. The last and final procedure follows 80% information for preparing and 20% information for testing. Table 2 shows these strategies for an assumed classifier. The following Figure 5 shows the proposed model architecture.

**Table 2:** Training and testing strategies with sample counts.

**Model**	**Classes**	**Total**	**S1:50-50**	**S2:60-40**	**S3:70-30**	**S4:80-20**
TL Model	Breast invasive carcinoma	878	439-439	527-351	615-263	702-176
	Kidney renal clear cell carcinoma	537	269-268	322-215	376-161	430-107
	Lung adenocarcinoma	162	81-81	97-65	113-49	130-32
	Lung squamous cell carcinoma	240	120-120	144-96	168-72	192-48
	Uterine corpus endometrial carcinoma	269	135-134	161-108	188-81	215-54

#### 3.4.2 Hyperparameter settings

Further, the Table 3 illustrates the pre-training parameters setting for comprehensive experimentation.

**Table 3:** Pre-training parameters for TL models.

**Sr#**	**Parameters**	**Values**
01	Learning rate (LR)	0.001
02	Batch size	64, 128, 256
03	Optimizer	Adam, SGDM
04	Momentum	0.9
05	L2 regularization	0.0001
06	Training and validation	80%–20%, 70%–30%, 60%–40%, 50%–50%
07	Number of epochs	100
08	Shuffle	Every epoch

## 4 Results and discussion

We summarize the discussion that involved characterization results for various cancer types.

### 4.1 Evaluation of model performance

In order to get an accurate estimate of the accuracy measure, we are using a variety of metrics. The measures include sensitivity, specificity, false positives (FP), false negatives (FN), true positives (TP), and true negatives (TN), as well as sensitivity (SEN), recall (REC), and accuracy (ACC) proportion. The term Delicacy is determined in Equation 2.


$$\begin{array}{l} \text{Sensitivity}\;\left(\text{SEN}\right)=\text{Recall}\;\left(\text{REC}\right)=\frac{\text{TP}}{\text{TP}+\text{FN}} \end{array}$$ (2)

Where TP is the counter for True Positive, and FN is the counter for False Negative. Pellucidity is called Specificity (SPE) and can be determined by Equation 3.


$$\begin{array}{l} \text{Specificity}\;\left(\text{SPE}\right)=\frac{\text{TN}}{\text{TN}+\text{FP}} \end{array}$$ (3)

ACC is determined to gauge the execution and implementation of the model, which can be determined as mentioned in Equation 4.


$$\begin{array}{l} \text{Accuracy}\;\left(\text{ACC}\right)=\frac{\text{TP}+\text{TN}}{\text{TP}+\text{TN}+\text{FP}+\text{FN}} \end{array}$$ (4)

False Positive Ratio (FPR) and False Negative Ratio (FNR) are presented in Equations 5, 6, respectively.


$$\begin{array}{l} \text{False Positive Rate}\;\left(\text{FPR}\right)=1-\text{True Negative Rate}\;\left(\text{TNR}\right) \end{array}$$ (5)

$$\begin{array}{l} \text{False Negative Rate}\;\left(\text{FNR}\right)=1-\text{True Positive Rate}\;\left(\text{TPR}\right) \end{array}$$ (6)

Where TNR and TPR are the rates of True Negative (Positive), respectively. Precision (PRE) and F1-scores are presented in Equations 7, 8, respectively.


$$\begin{array}{l} \text{Precision}\;\left(\text{PRE}\right)=\frac{\text{TP}}{\text{TP}+\text{FP}} \end{array}$$ (7)

$$\begin{array}{l} \text{F1-Score}=2\times \frac{\text{PRE}\times \text{REC}}{\text{PRE}+\text{REC}} \end{array}$$ (8)

### 4.2 Transfer learning-based classification

We have implemented five different TL models for similar examination among these methodologies without applying any increase or augmentation of data, and after applying augmentation to the information.

#### 4.2.1 Discussion for TL models without augmentation

**Figure 6:**
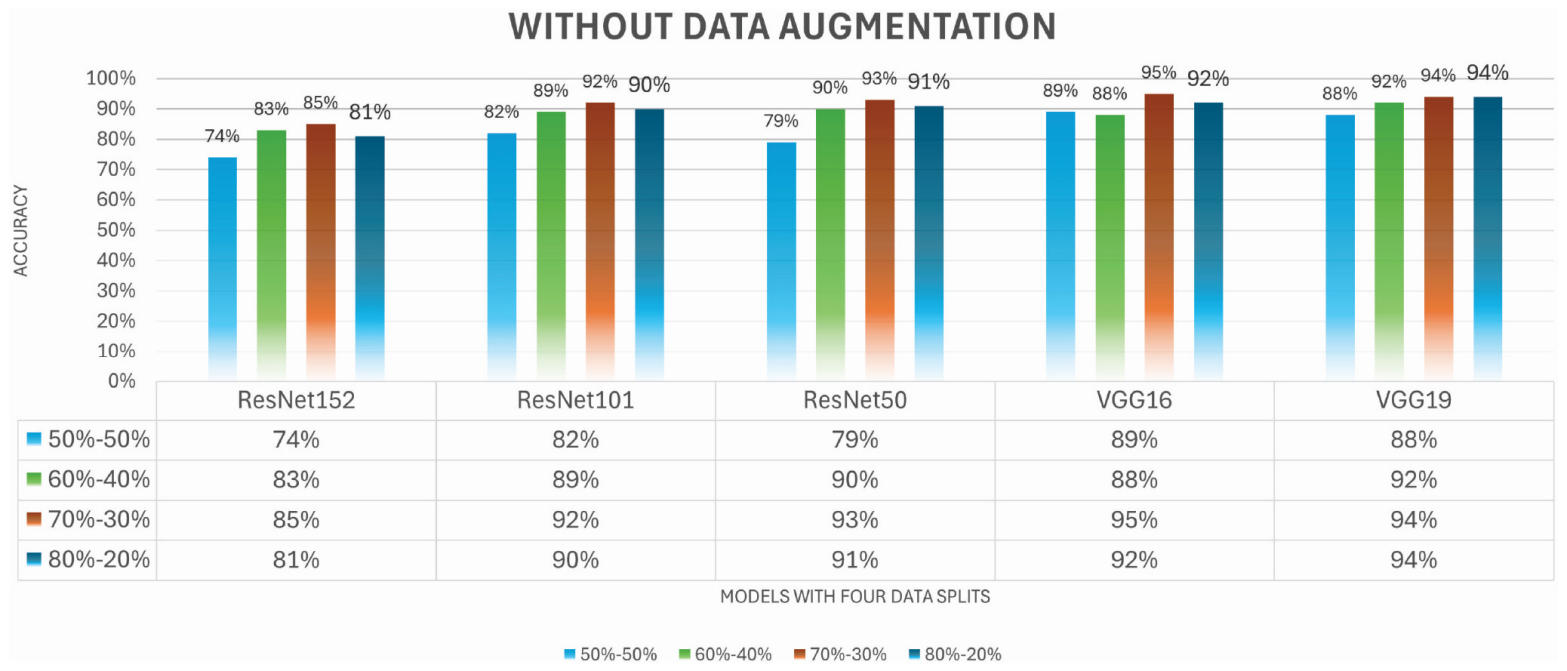
Comparative results for TL-based approaches without augmentation.

Figure 6 shows the accuracy of five TL models for cancer type classification with different splitting strategies without augmentation. TL models' performance for split 80-20, we analyzed that VGG19 outperformed all models at this split by achieving an accuracy of 94%. Performance of TL models for split 70-30 is analyzed, showing that VGG16 outperforms, gaining an increase in accuracy of 95%.

**Figure 7:**
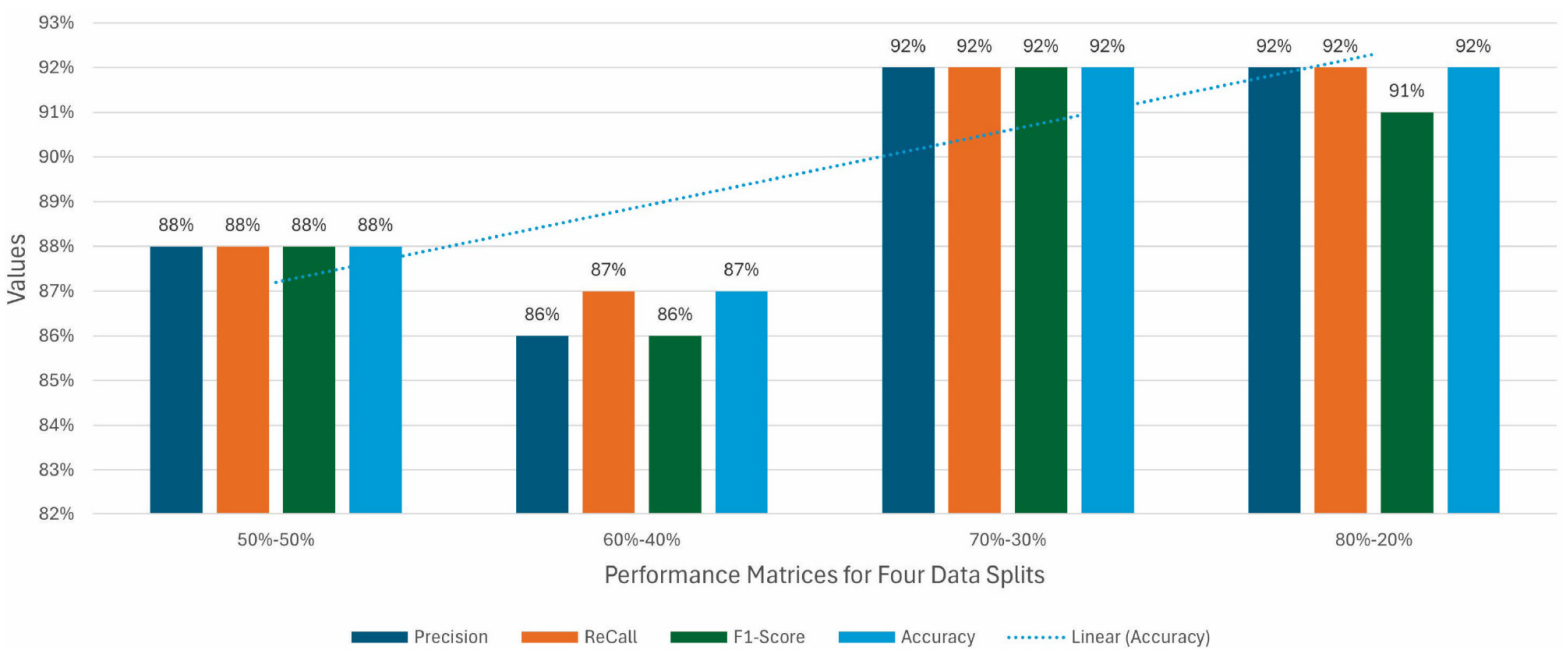
Precision, recall, F1 score, and accuracy for VGG16 without augmentation.

By reducing data in training, models suffer and decrease to 92% from 95%. VGG19 attained a high accuracy of 92% for a 60-40 split. For a 50-50 split, the less data in training shows an impact on the model's performance, and it can be easily noticed as the accuracy of each model decreases. VGG16 attained the best position by achieving 89% accuracy. Figure 7 illustrates all the performance matrices for the VGG19 model.

Furthermore, estimated macro-F1 score and estimated AUC scores are illustrated in Table 4. The analysis by the classes shows that the majority of errors happen within the clinically similar types of cancer or even among neighboring stages of severity, but not between biologically different classes. This implies that the borderline or phenotypically overlapping cases are the main challenge of the model. This type of confusion is clinically significant since falsely assigning a subtype to the related ones can influence treatment choices, and falsely assigning a subtype to ones at a distance is a relatively uncommon error.

**Table 4:** Best accuracy, with estimated macro-F1 and estimated AUC scores.

**Model**	**Accuracy**	**Estimated macro-F1**	**Estimated AUC**
ResNet152	85%	0.84	0.89
ResNet101	92%	0.91	0.95
ResNet50	93%	0.92	0.96
VGG16	95%	0.94	0.97
VGG19	94%	0.93	0.97

**Figure 8:**
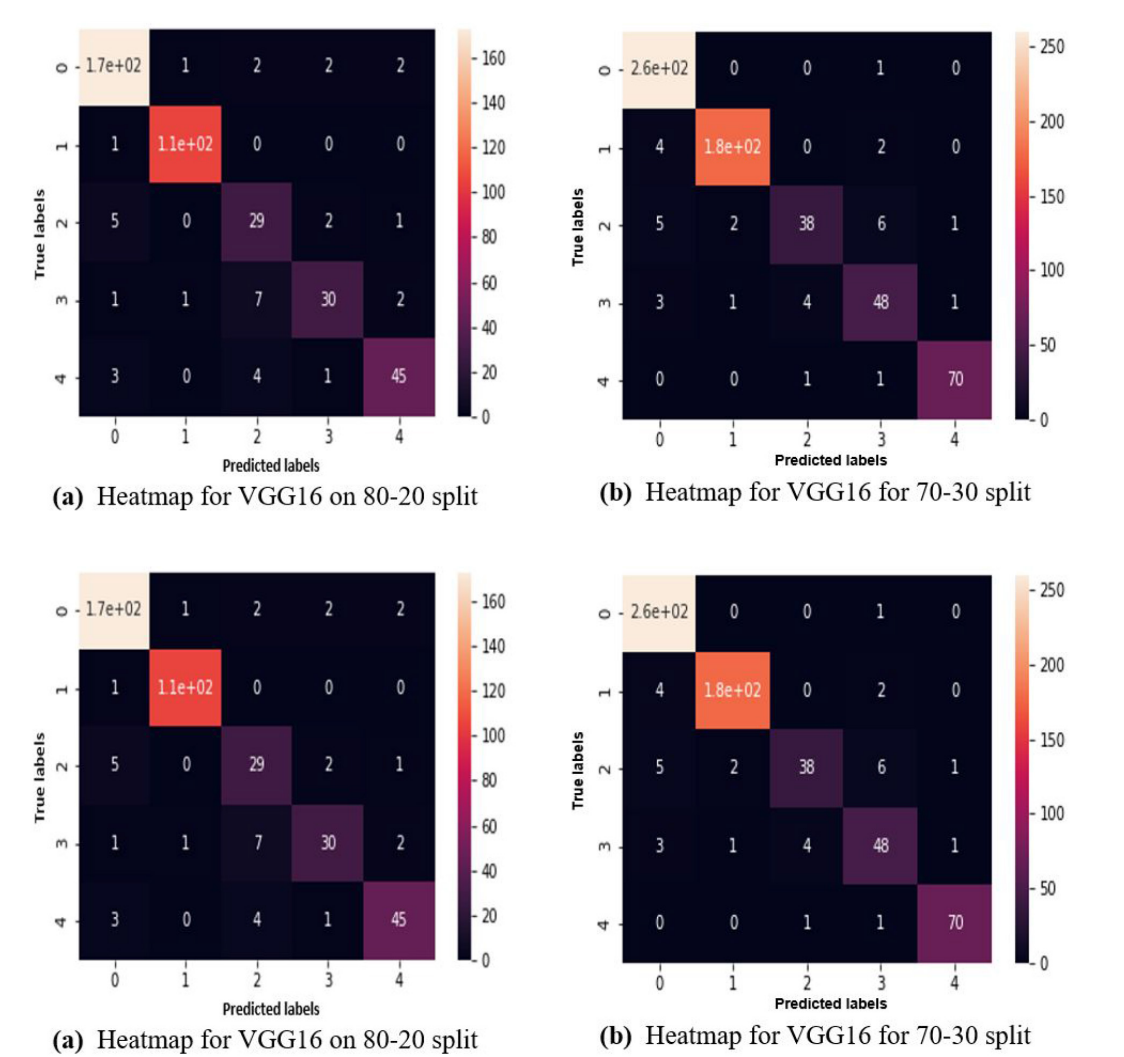
Heatmaps for VGG19 for **(a)** 80-20 split, **(b)** 70-30 split, **(c)** 60-40 split, **(d)** 50-50 split without augmented data.

Moreover, Figure 8a represents the heatmaps for the results of VGG19 on 50-50 data split, similarly, Figure 8b shows the heatmaps for the results of VGG19 on 60-40 data split, in continuation, Figure 8c shows the heatmaps for the results of VGG19 on 70-30 data split, finally, Figure 8d shows the heatmaps for the results of VGG19 on 80-20 data split, in the following representation.

Below Table 5 summarizes the performance of the transfer learning models across 70-30 train–test splits as shows relatively better performance. To assess statistical stability, results are reported as mean ± standard deviation across the selected data split. Among all models, VGG19 achieved the best overall performance with 94.16% ± 0.31, indicating both high accuracy and stable behavior across different data partitions. VGG16 followed closely with 95.0% ± 0.36, while ResNet50 and ResNet101 achieved comparable mean accuracies of 93.14 ± 0.28 and 92.06 ± 0.40, but with higher variance. ResNet152 showed the lowest performance at 85.08 ± 0.69. The lower standard deviation observed for the VGG-based models suggests more consistent generalization across varying splits.

**Table 5:** Mean and standard deviation values for all models on 70-30 split ratio.

**Model**	**Run1**	**Run2**	**Run3**	**Run4**	**Run5**	**Mean ± Std**
ResNet152	84.2	85.1	86.0	84.8	85.3	85.08 ± 0.69
ResNet101	91.5	92.3	92.1	91.8	92.6	92.06 ± 0.40
ResNet50	92.8	93.5	93.1	92.9	93.4	93.14 ± 0.28
VGG16	94.5	95.3	95.0	94.8	95.4	95.00 ± 0.36
VGG19	93.8	94.6	94.1	93.9	94.4	94.16 ± 0.31

#### 4.2.2 Discussion for TL models with augmentation

**Figure 9:**
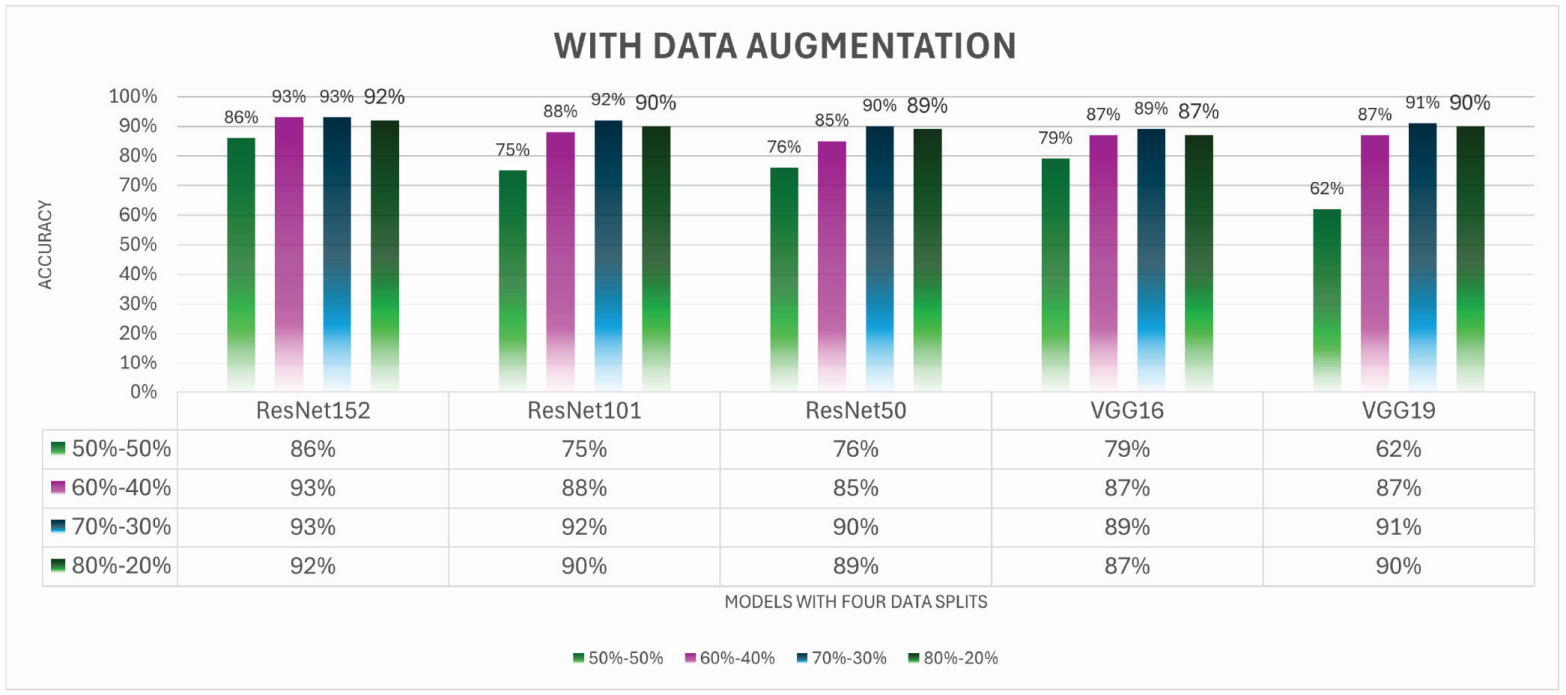
Comparative results for TL-based approaches with augmentation.

**Figure 10:**
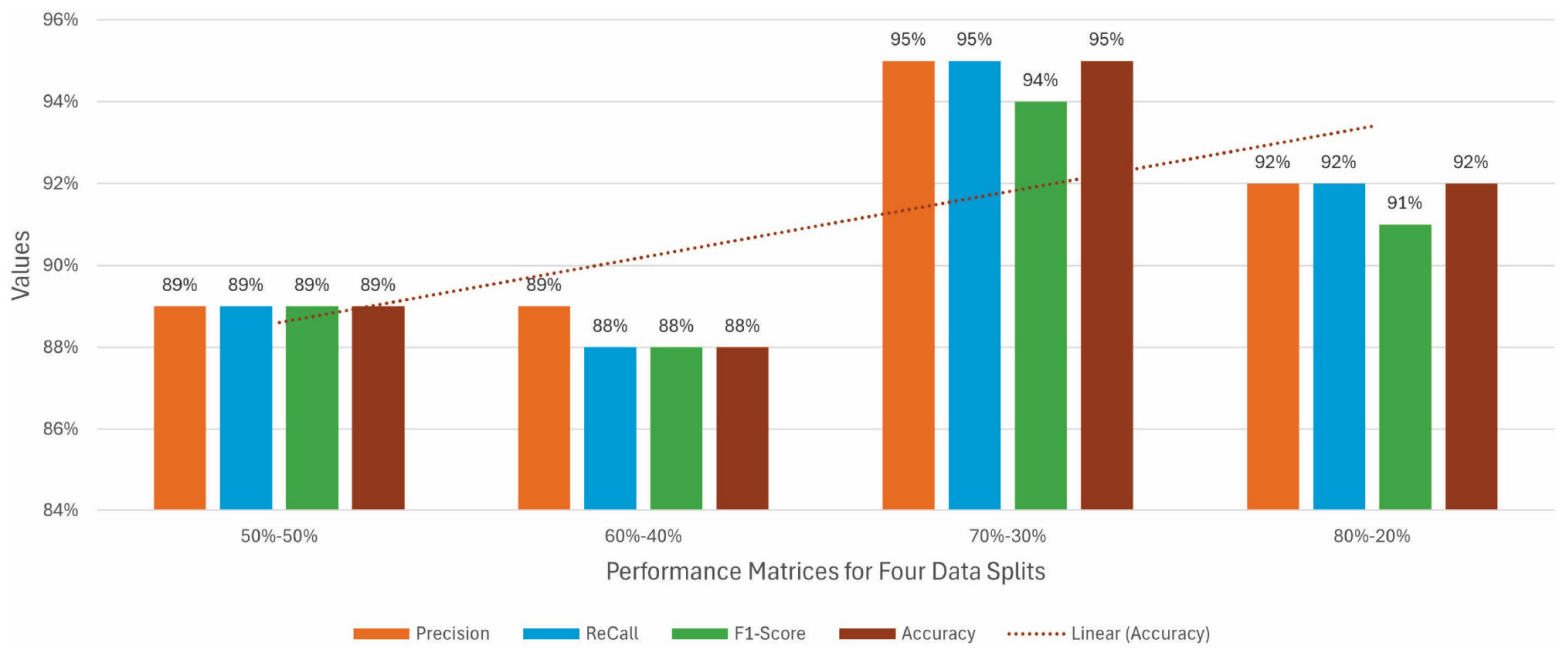
Precision, recall, F1 score, and accuracy for ResNet152 with augmentation.

The accuracy data presented in Figure 9 clarifies the similar aftereffects of these five different transfer learning calculations with expansion procedures applied, as examined before for expanding the size of the data and eliminating chances of overfitting. As a result of our investigation, which depicts a distribution of information for preparation and testing in the ratio of 80:20, we are able to conclude, without much of a stretch, that one classifier performed better than the others. ResNet models display correctness of 89%, whereas VGG designs display correctness of 87% and 89%. Further, Figure 10 illustrates all the performance matrices for the VGG19 model.

Both ResNet101 and VGG19 were able to outflank here with an exactness of 90% for this split. Here, it is simple to deduce that ResNet architectures improved their performance, and ResNet101 and ResNet152, which both achieve an accuracy of 92%, are the most effective of the bunch. The accuracies of the two different VGG designs are the same for the split 60-40 problem, which is 87%, while the accuracies of the three different ResNet architectures are 85%, 88%, and 87%, respectively. ResNet101 outperformed all TL approaches, as shown for split 60-40. The final accuracy report for the 50-50 split, where ResNet152 again gained its first position by achieving a high accuracy of 88%, and the accuracy of VGG19 architectures degraded to 62%.

Furthermore, estimated macro-F1 score and estimated AUC scores are illustrated in Table 6. Per-class analysis indicates that the majority of misclassifications are within cancer types that are both clinically or molecularly similar and not between biologically distinct classes. It means that the model has a major issue with borderline or overlapping phenotypes, which is also in line with the established transcriptomic similarity between related cancer subtypes. Clinically, these errors tend to be committed between related categories of disease, which may impact the stratification of treatment, but confusion between cancer types that are not closely related is relatively uncommon. This trend indicates that the model can represent general oncogenic signatures but might need extra class-specific features or imbalance-sensitive training to minimize clinically critical errors.

**Table 6:** Best accuracy, with estimated macro-F1 and estimated AUC scores on augmented dataset.

**Model**	**Accuracy**	**Estimated macro-F1**	**Estimated AUC**
ResNet152	93%	0.92	0.97
ResNet101	92%	0.91	0.96
ResNet50	90%	0.89	0.94
VGG16	89%	0.88	0.93
VGG19	91%	0.90	0.95

**Figure 11:**
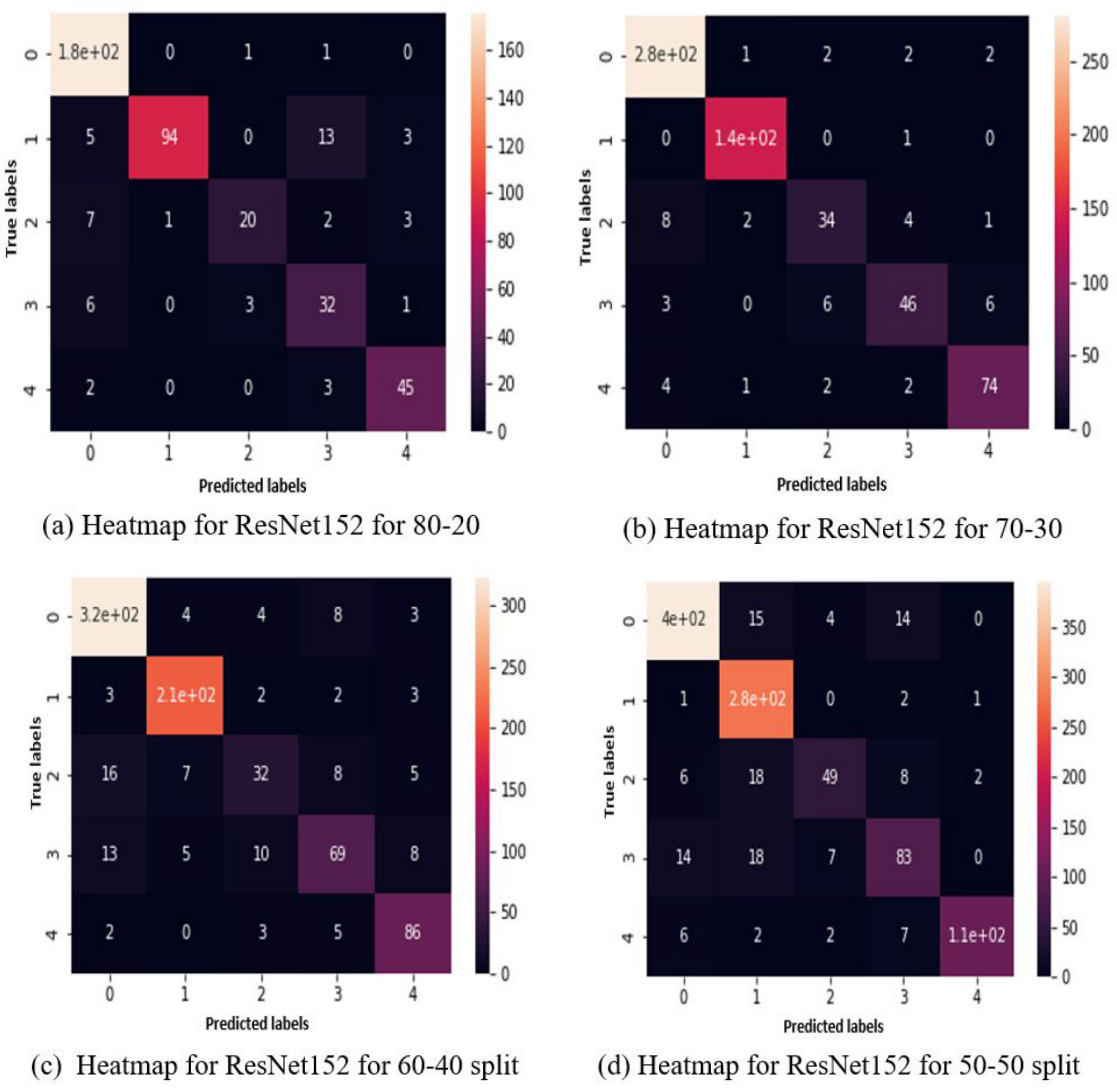
Heatmaps for ResNet152 for **(a)** 80-20 split, **(b)** 70-30 split, **(c)** 60-40 split, **(d)** 50-50 split with augmented data.

Above, Figure 11a represents the heatmaps for the results of ResNet152 on 50-50 data split with augmented data, similarly, Figure 11b shows the heatmaps for the results of ResNet152 on 60-40 data split with augmentation, in continuation, Figure 11c shows the heatmaps for the results of ResNet152 on 70-30 data split with augmentation, finally, Figure 11d shows the heatmaps for the results of ResNet152 on 80-20 data split with augmentation, in the following representation.

To evaluate statistical stability under data augmentation, we repeated the experiments on the 70-30 split across five independent runs using different random seeds, and report the results as mean ± standard deviation in Table 7. Among all models, ResNet152 achieved the highest performance with 93.12% ± 0.52, followed by ResNet101 with 92.16% ± 0.43. VGG19 obtained 90.96% ± 0.42, while ResNet50 and VGG16 showed slightly lower performance. The consistently low standard deviations across runs indicate stable and reliable model behavior under augmented training conditions.

**Table 7:** Mean and standard deviation values for all models on 70-30 split ratio with augmented data.

**Model**	**Run1**	**Run2**	**Run3**	**Run4**	**Run5**	**Mean ± Std**
ResNet152	92.4	93.2	93.7	92.8	93.5	93.12 ± 0.52
ResNet101	91.6	92.5	92.1	91.9	92.7	92.16 ± 0.43
ResNet50	89.4	90.2	90.6	89.8	90.3	90.06 ± 0.44
VGG16	88.5	89.4	89.1	88.8	89.6	89.08 ± 0.43
VGG19	90.4	91.3	91.0	90.6	91.5	90.96 ± 0.42

#### 4.2.3 Statistical ANOVA *Post-hoc* test

A one-way ANOVA across the five models showed a statistically significant difference in performance: *F* = 3.91, *p* = 0.0228 (! 0.05). Table 8. ANOVA showed that there was a significant difference between models in general (*p* = 0.0228). *Post-hoc* Tukey analysis indicated that VGG16 and VGG19 are significantly better in performance in comparison to ResNet152, ResNet101, and ResNet50, although there are no other significant differences between the pairs.

**Table 8:** ANOVA *Post-hoc* test across five TL models (significant comparisons only).

**Comparison**	**Mean difference**	***p*-value**	**Significant**
ResNet152 vs VGG16	10.25%	0.0368	Yes
ResNet152 vs VGG19	11.25%	0.0201	Yes

#### 4.2.4 Ablation study

In order to determine the contribution of the TL backbone, we tested a simple MLP classifier with the same input features and data splits. As it was always the case, the MLP demonstrated low performance in comparison to the TL models, proving that the suggested feature extraction strategy is effective. Results are represented in Table 9.

**Table 9:** Ablation study to compare TL models with MLP.

**Data**	**Model**	**S1**	**S2**	**S3**	**S4**
No augmentation	ResNet152	74%	83%	85%	81%
	ResNet101	82%	89%	92%	90%
	ResNet50	79%	90%	93%	91%
	VGG16	**89%**	88%	**95%**	92%
	VGG19	88%	**92%**	94%	**94%**
Augmentation	ResNet152	86%	93%	93%	92%
	ResNet101	75%	88%	92%	90%
	ResNet50	76%	85%	90%	89%
	VGG16	79%	87%	89%	87%
	VGG19	62%	87%	91%	90%
No augmentation	MLP	64%	55%	94%	94%

## 5 Conclusion and future direction

Transfer learning is a major advancement in AI and is replacing traditional methods for grouping and dimensionality reduction. It has implications for several real-world problems that people face. In this study, data from RNA-Seq were applied to five different forms of cancer. During the procedure known as “pre-processing,” the mathematical values are converted into 2D images. TL models utilize these pictures for classification. Five transfer learning models are used to classify multiple cancer types. The best outcome was achieved by VGG16 for the 70-30 split with an accuracy of 95%. Results are comparable to the techniques existing in the literature. In the future, the approach that was suggested could be improved to combine the processes of feature extraction and selection. Therefore, the data obtained from RNA-Seq could be put to use to identify illness biomarkers by carrying out pertinent research on genes that show promise.

## Data Availability

The original contributions presented in the study are included in the article/supplementary material, further inquiries can be directed to the corresponding authors.
